# Differential effects of heat shock protein 90 and serine 1179 phosphorylation on endothelial nitric oxide synthase activity and on its cofactors

**DOI:** 10.1371/journal.pone.0179978

**Published:** 2017-06-27

**Authors:** Yuanzhuo Chen, Bojie Jiang, Yugang Zhuang, Hu Peng, Weiguo Chen

**Affiliations:** Department of Emergency Medicine, Shanghai Tenth People’s Hospital, Tongji University School of Medicine, Shanghai, China; Max Delbruck Centrum fur Molekulare Medizin Berlin Buch, GERMANY

## Abstract

Endothelial nitric oxide synthase (eNOS) is responsible for maintaining systemic blood pressure, vascular remodeling and angiogenesis. Previous studies showed that bovine eNOS serine 1179 (Serine 1177 for human eNOS) phosphorylation enhanced NO synthesis. Meanwhile, heat shock protein 90 (Hsp90) plays a critical role in maintenance of eNOS structure and function. However, the regulatory difference and importance between Serine 1179 phosphorylation and Hsp90 on eNOS activity have not been evaluated. In current studies, S1179D eNOS was employed to mimic phospho-eNOS and exhibited markedly increased enzyme activity than wild type eNOS (WT eNOS). Hsp90 showed a dose-dependent increase for both WT eNOS and S1179D eNOS activity at the presence of all eNOS cofactors, such as Calcium/Calmodulin (Ca^2+^ /CaM), BH4, and NADPH etc. The enhancement effects were abolished by dominant-negative mutant Hsp 90 protein. ENOS-cofactors dynamic assay showed that Hsp90 enhanced WT eNOS affinity to NADPH, L-arginine, and CaM but not to Ca^2+^ and BH4. The impact of eNOS Serine 1179 phosphorylation and Hsp90 on eNOS affinity to cofactors has also been compared. Different from the effect of Hsp90 on eNOS affinity to specific cofactors, Serine 1179 phosphorylation significantly increased eNOS affinity to all cofactors. Moreover, VEGF-induced eNOS phosphorylation in bovine aortic endothelial cells (BAECs) and more NO generation from eNOS compared to control. Inhibition of Hsp90 by geldanamycin decreased eNOS activity and decreased endothelial viability. In conclusion, by changing eNOS structure, Hsp90 profoundly affected eNOS functions, including change of affinity of eNOS to cofactors like Ca^2+^, L-arginine, BH4 and further affecting NO generation capability. These specific cofactors regulated by Hsp 90 could become potential therapeutic targets of the eNOS-related diseases in future.

## Introduction

Endothelial nitric oxide synthase (eNOS) exists in various organs and tissue endothelium. ENOS converts L-arginine into L-citrulline and produces a high active molecule, nitric oxide (NO) which is a potent cell signaling and vasodilator molecule that plays important and diverse roles in biological processes, including control of vascular tone, vascular remodeling and angiogenesis [1]. NO generation from eNOS is under sophisticated and tight control. ENOS activity is not only regulated by its cofactors, such as Ca^2+^, CaM, L-arginine and BH4 [2], but also by its ammonia acid modifications, such as Serine 1177 and Threonine 495 phosphorylation (serine 1179 and Threonine 497 for bovine eNOS, respectively) [3–5]. VEGF and shear-stress mediated eNOS serine/threonine phosphorylation accompanied activation of eNOS and increase of NO production [6]. Mimicking the phosphorylation of Ser 1177 by mutation of Ser1177 to aspartic acid also enhances enzyme activity and alters the sensitivity of the enzyme to Ca^2+^ [7].

Heat shock protein 90 (Hsp90) is a chaperone protein that assists other cellular proteins to fold properly and stabilizes them against various adverse factors like oxidative or heat stress, as well as helping with protein degradation[8]. In endothelial cells, Hsp90 associates with eNOS and maintains eNOS normal structure and function [9–10]. Among all eNOS cofactors and chaperone proteins, heat shock protein 90 (Hsp 90) and BH4 have been proven as determinant switchers for eNOS generation of NO or superoxide (O^-.^_2_) [11–12]. By binding with eNOS oxygenase domain, Hsp90 keeps eNOS as an active form and inhibits superoxide generation. Inhibition of Hsp90 results in eNOS uncoupling and superoxide generation [11]. Yeast hybrid studies show that the M domain of Hsp90 (442–600) associates with eNOS oxygenase domain (2–403). This association is not only necessary for maintaining eNOS structure, but also for eNOS phosphorylation by Akt[13]. In contrast, Hsp90 inhibition may affect eNOS phosphorylation status and further affect eNOS activity [14]. Moreover, Hsp90α and Hsp90β have different effects on human eNOS Serine 1177 and Threonine 495 phosphorylation and on enhanced NO generation and superoxide generation in endothelial cells [15–16]. Compared to the importance of eNOS phosphorylation, Hsp90-mediated eNOS regulation and importance have not been completely elucidated.

In this study, using purified eNOS and cellular model, we determined the effect of Hsp90 on eNOS activity at basal and phosphorylated status. We also determined whether Hsp90 changes regulatory properties of eNOS cofactors, such as BH4, Ca^2+^, CaM. Our studies showed that Hsp90 increases eNOS activity by increasing eNOS affinity to L-arginine, CaM, and NADPH, but not to Ca^2+^ and BH4. On the other hand, Serine 1179 phosphorylation enhanced eNOS affinity to all eNOS cofactors, including L-arginine, NADPH, CaM/ Ca^2+^, and BH4. In contrast, Hsp90 did not change eNOS affinity to any cofactor at the condition of eNOS Serine 1179 phosphorylation.

## Materials and methods

### Materials

Bovine aorta endothelial cells (BAECs) and cell culture materials were obtained from Lonza (wakersville, MD). 2', 5’-ADP-Sepharose 4B was the product of Pharmacia Biotech. Inc. (Piscataway, NJ). L-[14C]-Arginine was purchased from DuPont/NEN (Boston, MA). N-methyl-D-glucamine dithiocarbamate/ferrous sulfate (MGD-Fe^2+^) were purchased from Enzo Life Science, Inc (Famingdale, NY). Purified Heat shock protein 90, Calcium Chloride, Calmodulin, NADPH, L-arginine, BH4, N-nitro-L-arginine methyl ester (L-NAME), and other reagents were purchased from Sigma Chemical Co. (St. Louis, MO), unless otherwise indicated. Wild type heat shock protein 90 (WTHSP90) and Dominant negative heat shock protein 90 (D88N-HSP90) plasmids were purchased from addgene (Cambridge, MA).

### ENOS purification

Recombinant bovine wild-type eNOS and S1179D eNOS plasmid were purchased from Addgene and overexpressed wild-type eNOS and S1179D eNOS in E.Coli. The expressed wild-type eNOS and S1179D eNOS proteins were harvested and purified according to the description of previous publications [17] [11].

### L-[^14^C]Arginine to L-[^14^C]Citrulline conversion assay

ENOS activity was determined by the L-[^14^C]arginine to L-[^14^C]citrulline conversion rate in a 200 μl of buffer containing 50 mM Tris-HCl, pH 7.4, 2μM L-[^14^C]arginine, 0.5 mM NADPH, 0.5 mM Ca2+, 10 μg/ml calmodulin, 2.5 μM BH4, and 5 μg/ml purified eNOS. After 5-min incubation at 37°C, the reaction was terminated by adding 3 ml of ice-cold stop buffer (20 mM Hepes, pH 5.5, 2 mM EDTA, 2 mM EGTA). L-[^14^C]Citrulline was separated by passing reaction mixtures through Dowex AG 50W-X8 (Na+ form, Bio-Rad) cation exchange columns and was quantitated by liquid scintillation counting [17].

### ENOS-mediated NADPH consumption measurement

ENOS consumption rate of NADPH was measured by absorption at 340 nm spectrophotometrically. The reaction systems were the same as described in EPR measurements, and the experiments were run at room temperature. The rate of NADPH oxidation was calculated using a molar extinction coefficient of 6.22 mM^-1^ cm^-1^ [12, 17].

### Measurement of intracellular NO generation in endothelial cells

For measurement of intracellular NO, Bovine aorta endothelial cells (BAECs) were plated in a 24 wells plate at a density of 2 × 10^4^ cells/ml and cultured with 20μM GA for 2days. After the cells were cultured with 50ng/ml VEGF for 20 minutes, the cells were washed with Dulbecco’s phosphate-buffered saline (DPBS) and then loaded with 1mM MGD-Fe2+. The NO generation from eNOS was measured by Fe2+-MGD-NO adduct using EPR with modification. The EPR recording parameters were: microwave frequency 9.8GHz, center field 3440G, modulation amplitude 6G, sweep width 100G, receiver gain 1×105, microwave power 10 mW, total number of scans 121, sweep time 10s, and time constant 20ms [11].

In the experiment of dominant negative heat shock protein 90 (D88N-Hsp90, DNHsp90) on eNOS activity, purified DNHsp90 plasmid or vector (pcDNA3.1) was transfected into BAECs using lipofectmine 2000. Next day, the transfection efficiency of DNHsp90 was determined by western blot [18]. And the NO generated from BAECs was measured by Fe2+-MGD-NO adduct using EPR as described[11].

### Cell viability analysis by 3-(4,5-dimethylthiazole)-2,5-diphenyl tetrazolium bromide (MTT) assay

BAECs were treated with 20μM geldanamycinn (GA) for 48 hours, and eNOS activated with 50ng/ml VEGF overnight. Following treatment, cellular viability was measured by MTT reduction assay, which is used to assess cell metabolic activity. 40 μl of 5 mg/ml MTT solution was added to each well. After 1 h of incubation, the supernatant absorbance of each well was measured spectrophotometrically at 570 nm (Cary 50 Bio, UV-Visible Spectrophotometer) according to the manufacturer's protocol [17].

### Statistics

Data was given as means ± SE. Student's *t*-test were used to compare means of data from two groups, whereas multiple group comparisons were tested by one-way ANOVA followed by Tukey’s Studentized range test. Differences were considered to be statistically significant at *P* < 0.05 or P<0.01.

## Results

### Serine 1179 aspartic acid mutation (S1179D) augments eNOS activity and increases eNOS sensitivity to Hsp90

Both wild type and S1179D eNOS were expressed and purified from E. coli. In a culture of 2 liters, the yield of eNOS was 2.5–4.0 mg. The purity of eNOS was identified on SDS gel using Coomassie staining. The activities of wild type and S1179D eNOS were measured by the conversion rate of L-[^14^C]-arginine to L-[^14^C]-citrulline. S1179D eNOS exhibited a two times higher conversion rate of L-[^14^C]-citrulline compared with wild type eNOS, which is consistent with our previous report and other group’s report (Data not shown)[4, 17].

**Fig 1:**
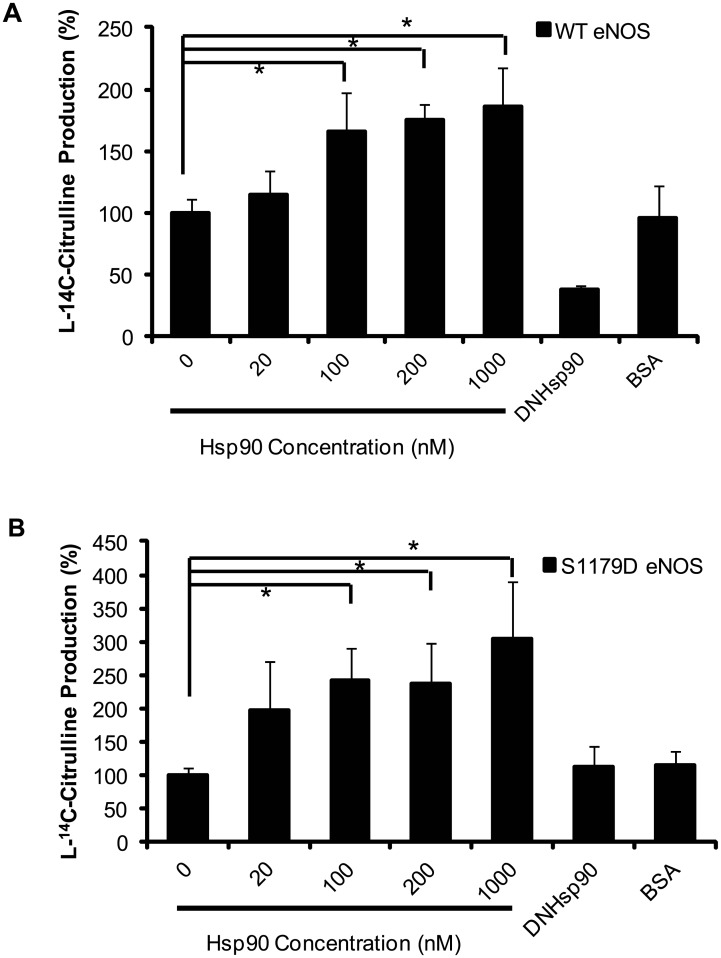
Hsp90 enhances both WT eNOS and S1179D eNOS activity. Purified WT eNOS and S1179D eNOS activities were assayed by monitoring the conversion of L-14C-arginine to L-14C-citrulline in the presence of different concentration of Hsp90. (A) Hsp90 enhanced WT eNOS as a dose-dependently and these enhancements were inhibited by dominant mutant Hsp90 (DNHsp90) (n = 5, *P<0.05, Hsp90 treatment groups compared to control). (B) Moreover, Hsp90 had higher enhancement effect on S1179D eNOS than it had on WT eNOS (n = 5, *, P<0.05 Hsp90 treatment groups compared to control).

Previous studies showed that Hsp90 has enhancement effect for eNOS activity [19–20]. But it is not clear whether Hsp90 also has different enhancement effects on WT eNOS and on phosphorylated eNOS. As shown in Fig 1A, Hsp90 has increased WT eNOS activity dose-dependently and activity reached a maximum at 100nM (1.6 times higher than control, Fig 1A WT eNOS). In contrast, eNOS activity was significantly blocked by dominant negative Hsp90 (DNHsp90), but was not affected by BSA(Fig 1A); Compared with WT eNOS, S1179D eNOS has significantly higher activity as same dosage of Hsp90 incubated with S1179D eNOS (2.5 times higher than control; Fig 1B S1179D eNOS). Compared to control and BSA groups, dominant negative mutant of Hsp90 (DNHsp90) significantly inhibited S1179D eNOS activity.

### Hsp90 enhances NADPH-mediated eNOS activity but only enhances WT eNOS combining sensitivity to NADPH

**Fig 2:**
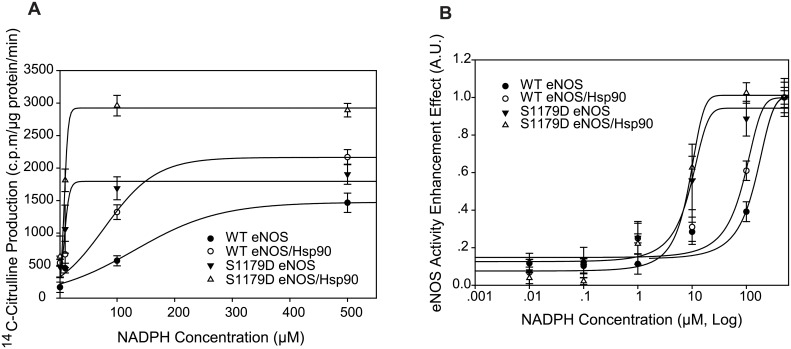
Hsp90 enhanced WT eNOS but not S1179D eNOS affinity to NADPH. (A) Hsp90 augmented both WT eNOS and S1179D eNOS activity in the presence of NADPH. The effect of Hsp 90 on enzymatic activity of WT eNOS and S1179D eNOS was assayed by monitoring the conversion of L-14C-arginine to L-14C-citrulline in the presence of indicated concentration of NADPH (n = 5). (B) NADPH-eNOS activity dynamic assay showed that Hsp90 enhanced eNOS affinity to NADPH in WTeNOS. In contrast, mutation of S1179D significantly enhanced eNOS affinity to NADPH in the absence and presence of Hsp90 compared to their control (EC50, P<0.05; n = 5).

In NOS-catalyzed reactions, the co-substrate NADPH is oxidized and serves as an electron donor for NO or O^-.^_2_ synthesis [2, 17]. Therefore, synchronous NADPH consumption always takes place accompanying NO or O^-.^_2_ generation. To investigate whether Hsp90 changes eNOS sensitivity to NADPH and NADPH consumption capability, eNOS activity was measured while incubating with indicated concentration of NADPH in the presence or absence of Hsp90. 100nM Hsp90 increased WT eNOS activity from 1466±148.2 c.p.m/μg protein/ min to 2168.3±116.5 c.p.m/μg protein/ min as it was incubated with 500μM NADPH. In contrast, 100nM Hsp90 enhanced S1179D eNOS activity from 1904.3±154.6 c.p.m/μg protein/ min to 2891.0±104.07 c.p.m/μg protein/ min as it was incubated with 100μM NADPH (Fig 2A). EC_50_ of NADPH consumption assay showed that Hsp90 shifted WTeNOS NADPH concentration from 133.1±21.1 μM to 82.5±14.2μM (Fig 2B); however, EC50 of Hsp90 mediated NADPH consumption did not significantly change in S1179D eNOS (8.9±0.12 μM to 7.7±0.14μM, Fig 2B). Compared to Hsp90, Mutation of Ser1179 to aspartic acid played a dominant role in changing eNOS sensitivity to NADPH.

### Hsp90 enhances L-Arginine-mediated eNOS activity but only enhances WT eNOS combining sensitivity to L-Arginine

**Fig 3:**
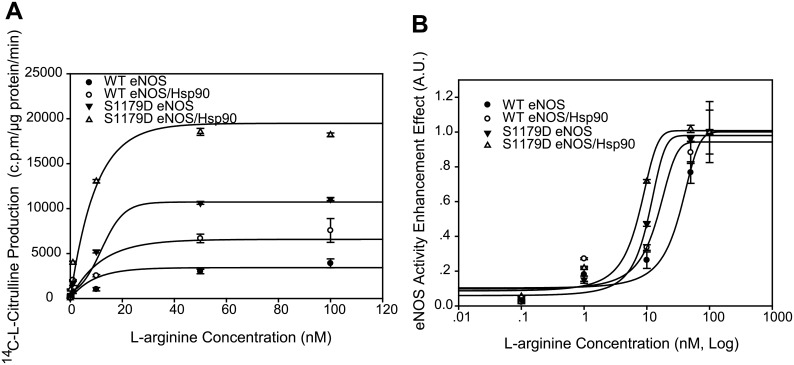
Hsp90 enhanced WT eNOS but not S1179D eNOS affinity to L-arginine. (A) Hsp90 augmented both WT eNOS and S1179D eNOS activity capacity in the presence of L-arginine (n = 5). (B) L-arginine -eNOS activity dynamic assay showed that Hsp90 enhanced WTeNOS (EC50, P<0.05; n = 5) but not mutant S1179D sensitivity to L-arginine (EC50, P>0.05; n = 5). However, mutation of S1179D significantly enhanced eNOS affinity to L-arginine compared to their WT eNOS control in the absence and presence of Hsp90 (EC50, P<0.05; n = 5).

As L-arginine is the substrate for eNOS NO generation [2], we then differentiated the effect of Hsp90 on L-arginine consumption by WT eNOS and S1179D eNOS. Hsp90 significantly increased L-arginine-mediated WT eNOS activity (from 3910.0±493.05 to 7570±1327.8 r.p.m/mg protein/min at 100nM L-arginine). Moreover, Hsp90 also significantly changed L-arginine binding affinity (EC50 WT eNOS vs WT eNOS /Hsp90 were 31.79±1.3 μM vs 15.28±0.2 μM, Fig 3A and 3B). S1179D eNOS had higher response to the same concentration of L-arginine comparing to WTeNOS (11060±160.37 c.p.m/μg protein/ min at 100nM L-arginine); Hsp90 further enhanced S1179D eNOS’s response to L-arginine (18200±211 c.p.m/μg protein/ min at 100nM L-arginine, Fig 3A). Mutation of Serine 1179 to aspartic acid slightly increased eNOS’s binding affinity to L-arginine (EC50 of S1179D eNOS vs S1179D eNOS/Hsp90 was10.06±0.4 μM vs 7.36±0.3 μM, Fig 3B).

### Hsp90 enhances calmodulin-mediated eNOS activity capacity but only enhances WT eNOS combining sensitivity to calmudolin

**Fig 4:**
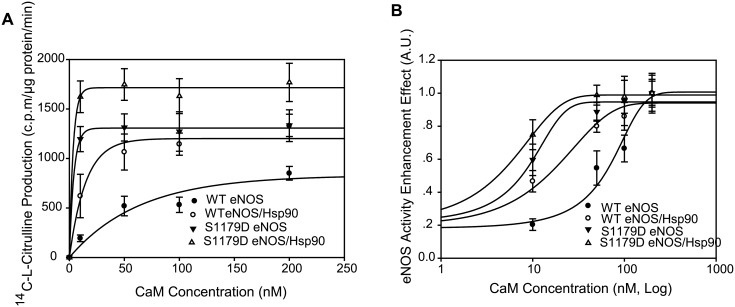
Hsp90 enhanced WT eNOS but not S1179D eNOS affinity to Calmodulin. (A) Hsp90 augmented both WT eNOS and S1179D eNOS activity capacity in the presence of calmodulin (n = 5). (B) Calmodulin -eNOS activity dynamic assay showed that Hsp90 enhanced WTeNOS (EC50, P<0.05; n = 5) but not mutant S1179D sensitivity to calmodulin (EC50, P>0.05; n = 5). However, mutation of S1179D significantly enhanced eNOS affinity to calmodulin compared to their WT eNOS control in the absence or presence of Hsp90 (EC50, P<0.05;n = 5).

In previous experiments, it was demonstrated that the "apparent calcium sensitivity" of eNOS was enhanced in cells expressing either a majority of phospho-eNOS or S1179D eNOS, suggesting that phosphorylation changed the affinity of calcium/CaM [7, 21]. [^14^C]-L-arginine to [^14^C]-L-citrulline conversion rate assay showed that Hsp90 significantly increased response capacity of both WT eNOS and S1179D eNOS to CaM (Fig 4A). The eNOS activity of WT eNOS in the absence and in the presence of Hsp90 were 850.5±69.2 and 1331.5±160.4 c.p.m/μg protein/ min at 200nM CaM, respectively. Similarly, the activities of S1179D eNOS in the absence and in the presence of Hsp90 were 1334.5±112.4 and 1768.17±193.12 c.p.m/μg protein/ min, respectively (Fig 4A). The CaM-eNOS affinity assay showed that Hsp90 significantly enhanced WTeNOS affinity with CaM; the EC50 values were 61.74±3.4 nM for WT eNOS alone and 15.4±1.6 nM for WT eNOS/Hsp90 (P< 0.05; Fig 4B). However, Hsp90 did not significantly enhance S1179D eNOS affinity with CaM; the EC50 values were 7.4±1.5 nM for S1179D eNOS alone and 4.31±1.2 nM for S1179D eNOS/Hsp90 (P> 0.05; Fig 4B).

### Hsp90 enhances calcium-mediated eNOS activity capacity but does not enhance either WT eNOS or S1179D eNOS combining sensitivity to calcium

**Fig 5:**
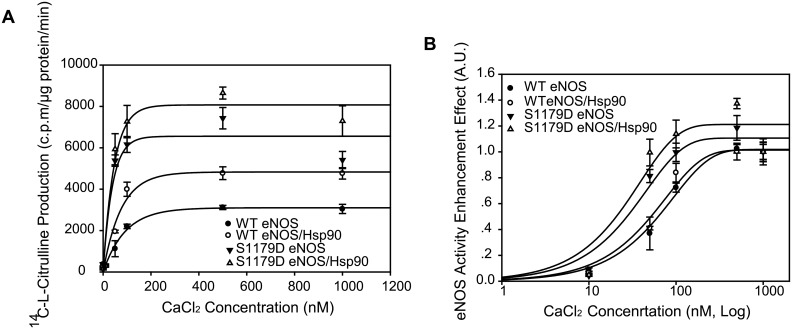
Hsp90 did not enhance either WT eNOS or S1179D eNOS affinity to calcium. (A) Hsp90 augmented both WT eNOS and S1179D eNOS activity capacity in the presence of calcium. The effect of Hsp 90 on enzymatic activity of WT eNOS and S1179D eNOS was assayed by monitoring the conversion rate of L-14C-arginine to L-14C-citrulline in the presence of indicated concentration of calcium chloride (n = 5). (B) Calcium chloride -eNOS activity dynamic assay showed that Hsp90 did not change either WTeNOS or mutant S1179D sensitivity to calcium ((EC50, P>0.05; n = 5). On the other hand, mutation S1179D significantly enhanced eNOS affinity to calcium comparing to their WT eNOS control in the absence or presence of Hsp90 (EC50, P<0.05; n = 5).

Similar with the effect of Hsp90 on response of eNOS to CaM, Hsp90 caused significantly higher eNOS activity for both WT eNOS and S1179D eNOS. Incubation of Hsp90 with WTeNOS enhanced its activity from 3117.5±100.4 c.p.m/μg protein/ min to 4772.7±308.8 c.p.m/μg protein/ min for WT eNOS (at 500nM Ca^2+^); similarly, Incubation of Hsp90 with S1179D eNOS enhanced its activity from 7431.2±571.01 c.p.m/μg protein/ min to 8654.7±289.8 c.p.m/μg protein/ min (at 500nM Ca^2+^, Fig 5A). The Ca^2+^-eNOS affinity assay showed that Hsp90 did not significantly enhance WTeNOS affinity to Ca^2+^; the EC50 values were 65.4±7.6 nM for WT eNOS alone and 53.1±5.4 nM for WT eNOS/Hsp90 (P> 0.05; Fig 5B). Similarly, Hsp90 also did not significantly enhance S1179D eNOS affinity with Ca^2+^; the EC50 values were 27.5±2.7 nM for S1179D eNOS alone and 21.2±5.1 nM for S1179D eNOS/Hsp90 (P> 0.05; Fig 5B).

### Hsp90 enhances BH4-mediated eNOS activity capacity but does not enhance either WT eNOS or S1179D eNOS combining sensitivity to BH4

**Fig 6:**
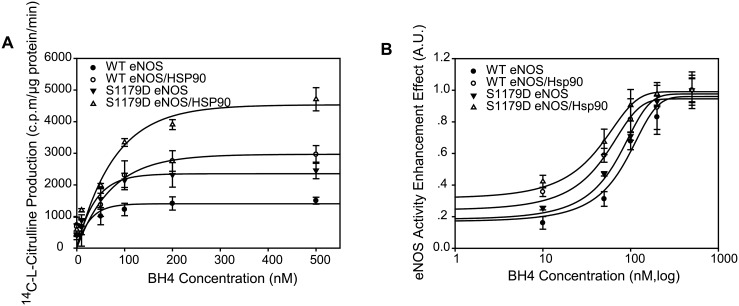
Hsp90 did not enhance either WT eNOS or S1179D eNOS affinity to BH4. (A) Hsp90 augmented both WT eNOS and S1179D eNOS activity capacity in the presence of BH4. The effect of Hsp 90 on enzymatic activity of WT eNOS and S1179D eNOS was assayed by monitoring the conversion rate of L-14C-arginine to L-14C-citrulline in the presence of indicated concentration of BH4 (n = 5). (B) BH4-eNOS activity dynamic assay showed that Hsp90 did not change either WTeNOS or mutant S1179D sensitivity to BH4 (EC50, P>0.05; n = 5). In contrast, mutation of S1179D significantly enhanced eNOS affinity to BH4 compared to their WT eNOS control in the absence or presence of Hsp90 (EC50, P<0.05;n = 5).

To investigate whether Hsp90 changed the effect of BH4 on eNOS, BH4-dependent eNOS activities were evaluated in the presence or absence of Hsp90. As seen in Fig 6A, BH4 demonstrated a dose-dependent increase of WT eNOS activity and reached a maximum at 500nM BH4. Hsp90 treatment increased WT eNOS activity capacity from 1499.8±109.4 c.p.m/μg protein/ min to 2962.5±281.2 c.p.m/μg protein/ min. Similarly, Hsp90 treatment increased S1179D eNOS activity from 2457.8±261.7 c.p.m/μg protein/ min to 4707.6±366.5 c.p.m/μg protein/ min (Fig 6A). Meanwhile, BH4-mediated eNOS activity dynamic curves assay showed that Hsp90 treatment did not significantly change EC50 of BH4 for eNOS activity compared to control. For WT eNOS, EC50 of BH4 were 79.13±17.5 nM and 60.1±11.2 nM (P> 0.05; Fig 6B) for control and Hsp90 treatment, respectively. For S1179D eNOS, EC50 of BH4 were 38.6±8.7 nM and 26.8±7.5 nM (P> 0.05; Fig 6B) for control and Hsp90 treatment, respectively.

### Hsp90 enhances eNOS activity at both basal and phosphorylated condition in BAECs

**Fig 7:**
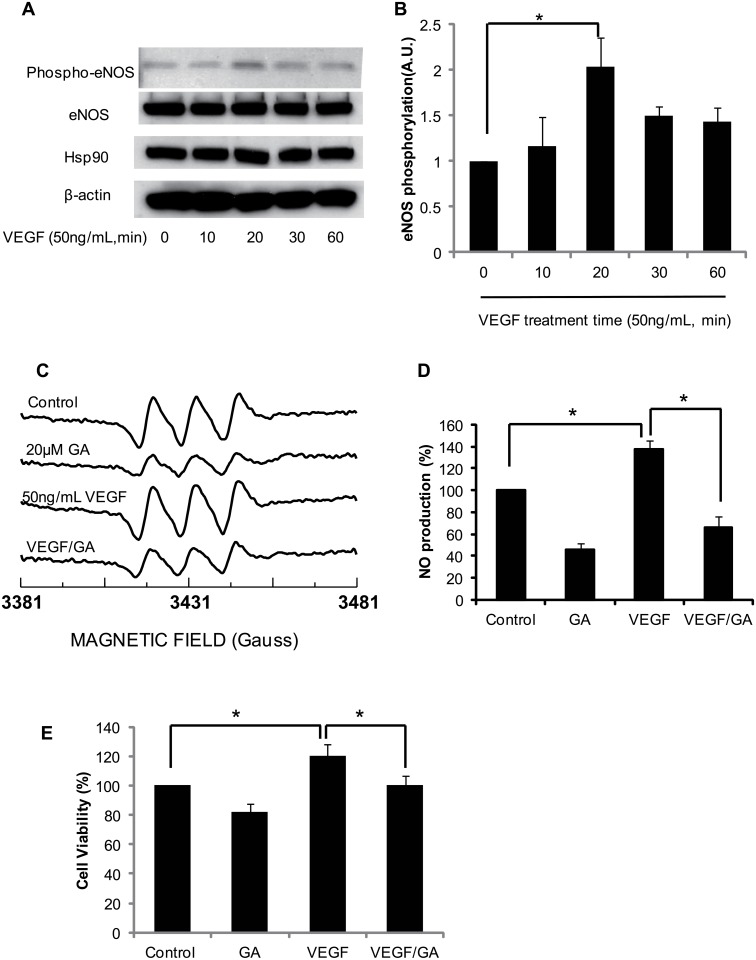
Inhibition of Hsp90 abolished VEGF-mediated eNOS phosphorylation and decreased NO production and cell viability in BAECs. (A and B) BAECs were treated 50ng/mL VEGF indicated time. The cell lysates were harvested for western blot using indicated antibodies (*, P>0.05, compared to control; n = 3). (C and D) In different experiments, BAECs were treated with 50ng/mL VEGF in the absence or presence of geldanamycin. After adding 1mM MGD for 30minutes, the medium was harvested for NO production measurement using EPR(*, P>0.05, VEGF group compared to control or VEGF compared to VEGF/GA group; n = 5). (E) BAECs were treated with VEGF after incubated with geldanamycin for 2 days, the cells were incubated with MTT and the cells viability was measured as described in methods (*, P>0.05, VEGF group compared to control or VEGF compared to VEGF/GA group; n = 5).

Previous studies showed that VEGF caused eNOS Ser1177 phosphorylation accompanying eNOS activity to increase [5]. Consistent with these observations, VEGF caused eNOS S1177 phosphorylation in time-dependent way and reached to maximum at 20min in BAECs (Fig 7A). To characterize the effect of Hsp90 on eNOS activity, BAECs were treated with 20μM geldanamycin. After treatment of geldanamycin for 2 days, the cells were starved for 4 hours and followed by 50ng/mL VEGF challenged. The medium, which incubated with 1mM MGD, was harvested for EPR. EPR density assay showed that a strong MGD-NO adduct signal was observed in control group cells. 20μM geldanamycin treatment significantly inhibited the MGD-NO adduct signal generated from cells. EPR signal showed a significant augment of the MGD-NO adduct signal in BAECs as they were treated with VEGF. The VEGF-mediated MGD-NO adduct signal augment was attenuated by geldanamycin (Fig 7C and 7D). To further define the importance of Hsp90 on eNOS and cell fate, BAECs were incubated with 20μM geldanamycin in the presence and absence of VEGF. 20μM geldanamycin alone caused a significant cell death rate in BAECs. Moreover, geldanamycin also significantly inhibited VEGF-mediated cell growth and differentiation (Fig 7E).

### Dominant negative Hsp90 (DNHsp90) inhibits eNOS serine 1179 phosporylation and activity in BAECs

**Fig 8:**
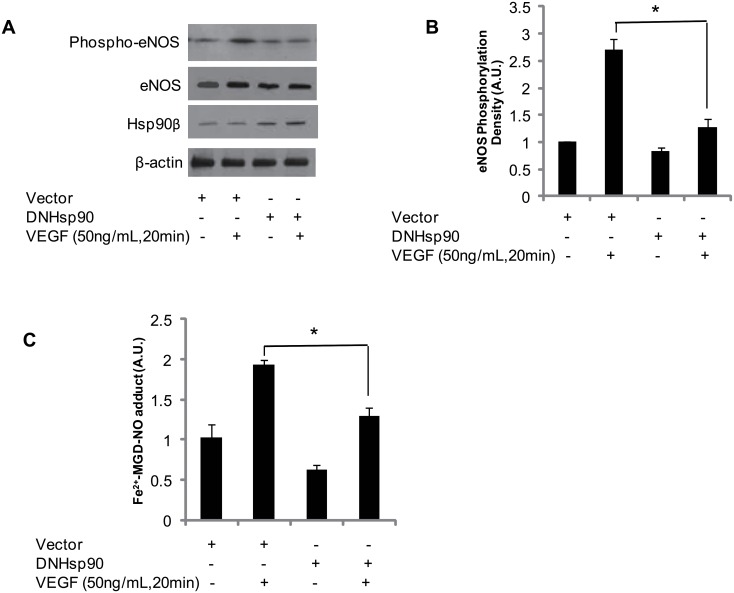
Dominant negative Hsp90 inhibited VEGF-mediated eNOS phosphorylation and NO generation from BAECs. (A and B) Overexpression of DNHsp90 in BAECs increased Hsp90β expression and significantly inhibited VEGF-mediated eNOS phosphorylation (P<0.05 compared to control; n = 4); (C) Fe2+-MGD-NO adduct signal density assay showed that DNHsp90 also significantly inhibited VEGF-mediated NO generation from BAECs (P<0.05 compared to control; n = 6).

Previous studies showed that dominant negative Hsp90 (Aspartate 88 mutates (D) to Asparagine (N), DNHsp90) interfered with the endogenous Hsp90β and Hsp90α, further affecting the complex stability of Hsp90, eNOS and Akt[18]. To evaluate the importance of Hsp90 for eNOS function, dominant-negative Hsp90 (DNHsp90) or vector (pcDNA 3.1) were transfected into BAEC. eNOS phosphorylation and NO generation from BAECs were evaluated. Overexpression of DNHsp90 slightly decreased eNOS content in BAEC, but significantly inhibited VEGF-mediated eNOS phosphorylation (Fig 8A and 8B). Moreover, DNHsp90 also significantly inhibited VEGF-induced NO generation in BAECs (Fig 8C). These observations are consistent with previous reports [18].

## Discussion

In this study, we tested the hypothesis that heat shock protein 90 (Hsp90), a chaperone protein, regulates nitric oxide generation from eNOS and that Hsp90 can also influence the sensitivity of eNOS to multiple regulatory cofactors. Moreover, we also characterized the regulatory properties of Hsp90 on eNOS while eNOS was under phosphorylated conditions. We found that Hsp90 significantly enhanced eNOS activity of wild type eNOS and phosphomimetic mutant S1179D eNOS. We also found that Hsp90 significantly increased the NADPH, L-arginine, and CaM sensitivity, while not significantly changing the Ca^2+^, BH4 sensitivity to eNOS. As eNOS was under serine 1179 phosphorylated condition, Hsp90 did not significantly change any cofactor’s affinity to eNOS. In contrast, Serine 1179 phosphorylation significantly increased both non-Hsp90-binding eNOS and Hsp90-binding eNOS’s affinity to all eNOS cofactors, including Ca^2+^, BH4, L-arginine, NADPH, and CaM.

As a chaperone protein, Hsp90 associates with all three different NOSs. Structure studies indicate that Hsp90 associates with the oxygenase domain of eNOS in which eNOS also associates with many kinds different cofactors, such as Heme, BH4, and L-arginine[22]. There are two major ways to regulate eNOS activity through proteins association changes. The first way is that CaM replaces caveolin and dephosphorylates Threonine 497, which results in eNOS activation [23]. The second way is that Hsp90 associates with eNOS and recruits AKT, which causes Serine 1177 phosphorylation and enhances eNOS activity[24]. In this in vitro experiment, we found that Hsp90 enhances purified eNOS activity; meanwhile it also enhances eNOS affinity to NADPH, L-arginine, and CaM. While our eNOS activity assay system lacks AKT, the regulatory mechanism of Hsp90 on eNOS activity and on affinity to NADPH, L-arginine, and CaM is unlikely to be explained exclusively by AKT-mediated Serine 1179 phosphorylation.

ENOS Serine 1177 phosphorylation (Serine 1179 for bovine eNOS) has been intensively studied and is a very important regulatory mechanism for eNOS activity. Serine 1177 phosphorylation can enhance eNOS NO generation or superoxide generation capacity [25–26]. Consistent with previous studies, we found that Serine 1179 phosphorylation enhanced eNOS activity in the absence or presence of Hsp90. Moreover, Serine 1179 phosphorylation also enhanced eNOS affinity to all cofactors compared to WT eNOS control. These results indicate that Serine 1179 phosphorylation-mediated eNOS activity enhancement mechanismis different from Hsp90. More interestingly, Hsp90 did not significantly cause eNOS affinity changes to all cofactors at Serine 1179 phosphorylated conditions. These results indicate that Serine 1179 phosphorylation may play a dominant role in regulation of eNOS affinity to cofactors compared to Hsp90.

As many proteins associate with eNOS, elucidation of the regulatory mechanisms of these associated proteins helps with understanding eNOS function. In eNOS oxygenase domain, Hsp90 (442–600 aa) associates with eNOS (300-400aa). In the rest of the conditions, caveolin-1 binds with eNOS oxygenase domain (350-358aa) and binds other cofactors such as CaM, binding to eNOS [11, 22]. At certain conditions, such as VEGF stimulation, AKT binds with Hsp90 (327-340aa) and phosphorylates eNOS Serine 1179[3, 19]. Caveolin and Hsp90 may keep balance between activation and inactivation of eNOS [27]. In current studies, rather than enhancing all eNOS Cofactors affinity by Serine 1179 phosphorylation, Hsp90 specifically changes a few cofactors affinity, such as NADPH, L-arginine, and CaM. These changes indicate that Hsp90 regulates eNOS in unique way and could be adopted by clinic purposes.

In summary, both Hsp90 and Serine 1179 phosphorylation enhance eNOS activity. Hsp90 enhances eNOS affinity to NADPH, L-arginine, and CaM, but not to Ca^2+^ and BH4. However, Serine 1179 phosphorylation enhances eNOS affinity to all cofactors. Moreover, Hsp90 does not affect eNOS affinity to any cofactor at the condition of eNOS Serine 1179 phosphorylation. Inhibition of Hsp90 causes eNOS NO generation and cell viability decreases. These novel findings advance our understanding of eNOS regulation, and offer new insight into the mechanisms underlying eNOS dysfunction and related clinical disorders.

## Abbreviations

BAECs: Bovine aorta endothelial cells
BH4: tetrahydrobiopterin
Ca^2+^: Calcium ion
CaM: Calmodulin
DNHsp90: Dominant negative (D88N) heat shock protein 90
eNOS: endothelial NOS
EPR: electron paramagnetic resonance
FPLC: Fast Performance Liquid Chromatography
GA: geldanamycin
Hsp 90: heat shock protein 90
L-NAME: *N*-nitro-L-arginine methyl ester
MGD-Fe2+: N-methyl-D-glucamine dithiocarbamate
MTT: 3-(4,5-dimethylthiazole)-2,5-diphenyl tetrazolium bromide
NO: nitric oxide
NOS: NO synthase
O^-.^_2_: superoxide
ROS: reactive oxygen species
S1179D eNOS: Serine 1179 aspartic acid eNOS mutant
VEGF: vascular endothelial growth factor
WT eNOS: wild type eNOS
